# Impact of Physical Exercise Programs and Programs of Social Activity on Public Health and Social Inclusion of Young People

**Published:** 2019-06

**Authors:** Bojan MASANOVIC

**Affiliations:** Faculty for Sport and Physical Education, University of Montenegro, Podgorica, Montenegro

## Dear Editor-in-Chief

Being socially included means being able to fully and productively participate in economic, social and cultural life, and also to enjoy the standard of living, that is, the well-being that is considered normal in a developed community (1). Therefore, if we start from the fact that three dimensions of social involvement are established (2) and that they are related to friendship, the feeling of personal value and optimism (3), we conclude that a person cannot be considered socially included unless he is able to exercise friendship experience, perform useful and meaningful activities in society, and hope for a better future. Therefore, if we know that physical activity positively influences physical, psychological, social and professional aspects (2), and if we know from previous research that it can positively influence public health, both mental and physical aspect (4), then we must use it.

The aim of this research was to determine whether organized physical exercise can be better than some other social activity, to influence the level of social inclusion of young people. This study included a sample of 60 respondents aged between 18 and 26 yr, divided into three groups: the first experimental group of 15 respondents for which a 3-month experimental physical exercise program was organized. The second experimental group of 15 respondents, for which a social gathering was organized; third group, a control group of 30 subjects who performed normal activities.

The criterion for inclusion in the experiment was that respondents are older than 18 and under the age of 26, that they have no health problems, that they have never been active in sports, and that they do not engage in any sport activities for a longer period of time. Exercises that were used in practical work were adapted to the abilities of the respondents, and focused on acquiring general physical preparedness. A research technique to determine the degree of social inclusion was a survey in which a standard questionnaire called the “Social Inclusion Scale” was used, consisting of 18 questions with the social inclusion scale having three subcategories that measure social isolation, social relationships and social acceptance (5).

Informed consent was taken from the participants before the study.

Empirical data were analyzed through SPSS 20.0 (Chicago, IL, USA). ANOVA and MANOVA were used to determine the significance of the differences in the degree of social inclusion between the groups at the initial measurement, and also after the end of the experimental treatment. The significance was set at an alpha level of 0.05.

The results of this study showed that the application of experimental work programs did not contribute to significant progress of respondents when it comes to social inclusion. Although the experimental physical exercise program lasted 3 months, significant progress in relation to the initial state was not made for even a single system of subcategories (social isolation, social relations, and social acceptance). For both the initial and final measurement, the difference was found only in question number 10 (F=4.028, p=.023 - initial measurement; F=3.749, *P*=0.30 - final measurement). However, if we only look at the group that practiced according to the experimental program, we can notice that there are slightly more positive answers for each question from the social acceptance subgroup.

On the basis of this, we can conclude that the exercise program, however, in some way contributed to the fact that respondents felt more socially accepted. We cannot make this conclusion for the members of the other two groups of respondents. Previous research clearly indicates that there is a positive impact of activities such as sports and recreation on social inclusion (4, 6). However, they do not provide accurate data on the statistical significance of the sport’s impact. On the other hand, there are studies suggesting that any organized activity that has the effect of suppressing loneliness (7, 8) positively affects the social involvement of an individual, regardless of whether it is sport or any other social engagement.

However, the advantage of sport can be that in addition to achieving social inclusion, participants also get a positive impact on the health and quality of life, which would again mean that they have solved two problems with one move. The limitation of this study is that the sample of the respondents is not large enough to generalize the conclusion, also in the fact that the experimental program is insufficiently long to cause a change, therefore the next examination should focus on a larger group of respondents, and the collection of data on social exclusion of persons who in the long run deal with sports activities.
